# Quantifying Infarct Growth and Secondary Injury Volumes: Comparing Multimodal Image Registration Measures

**DOI:** 10.1161/STROKEAHA.118.020788

**Published:** 2018-06-12

**Authors:** George W.J. Harston, Davide Carone, Fintan Sheerin, Mark Jenkinson, James Kennedy

**Affiliations:** 1 From the Radcliffe Department of Medicine, Acute Vascular Imaging Centre (G.W.J.H., D.C., F.S., J.K.); 2 Nuffield Department of Clinical Neurosciences, Wellcome Centre for Integrative Neuroimaging (M.J.), University of Oxford, United Kingdom.

**Keywords:** edema, follow-up studies, humans, magnetic resonance imaging, stroke, tomography, X-ray computed

## Abstract

**Background and Purpose—:**

Lesion expansion in the week after acute stroke involves both infarct growth (IG) and anatomic distortion (AD) because of edema and hemorrhage. Enabling separate quantification would allow clinical trials targeting these distinct pathological processes. We developed an objective and automated approach to quantify these processes at 24 hours and 1 week.

**Methods—:**

Patients with acute ischemic stroke were scanned at presentation, 24 hours, and 1 week in a magnetic resonance imaging (MRI) cohort study. IG and AD were calculated from follow-up lesion masks after linear and nonlinear registration to a presenting MRI scan. Performance of IG and AD was compared with edema quantified using cerebrospinal fluid displacement. The use of alternative reference images to define AD, including template MRI, mirrored MRI, and presenting computed tomographic scan, was explored.

**Results—:**

Thirty-seven patients with nonlacunar stroke were included. AD was responsible for 20% and 36% of lesion expansion at 24 hours (n=30) and 1 week (n=28). Registration-defined IG and AD compared favorably with edema quantified using cerebrospinal fluid displacement, particularly at smaller infarct volumes. Presenting computed tomographic imaging was the preferred alternative reference image to presenting MRI for measuring AD.

**Conclusions—:**

The contributions of IG and AD to lesion expansion can be measured separately over time through the use of image registration. This approach can be used to combine imaging outcome data from computed tomography and MRI.

## Abstract 2

Supplemental Digital Content is available in the text.

With the widespread adoption of mechanical thrombectomy into clinical practice, there is renewed interest in strategies to mitigate secondary injury for patients with acute ischemic stroke, including neuroprotection and minimizing vasogenic edema.^1,2^ Lesion expansion after the ischemic insult is because of a combination of infarct growth (IG), which is associated with poor long-term clinical outcomes^3,4^ and anatomic distortion (AD) because of cerebral edema and hemorrhagic transformation—the major cause of neurological deterioration and death in the days after the event.^5–7^ Quantifying IG and AD separately poses significant challenges and limits the opportunity for clinical trials to assess treatment efficacy.

The most common approaches to defining IG in trials have used either differences in measured infarct volumes between time points or the identification of regions of new infarction after linear image registration.^8,9^ Both will be confounded by AD because of edema or hemorrhage included in the volume. To minimize confounding, IG has been defined using extension into new anatomic territories.^10,11^ However, moving away from a volume-based approach compromises the ability to demonstrate a potential treatment effect.

Quantification of edema is similarly challenging. Current strategies involve labor intensive and subjective methodology defining regions on a slice-by-slice basis,^12–14^ measures of midline shift insensitive to submassive distortions,^2^ or inferences about focal edema from changes in whole brain or cerebrospinal fluid (CSF) volumes, which have produced inconsistent results and a fixed error that limits their use in smaller infarcts.^15,16^ Despite these challenges, the importance of AD has been highlighted by manual quantification of brain swelling, which has identified >11 mL volume as the threshold with greatest sensitivity and specificity for predicting poor outcome.^11^

Image registration has been key in the interpretation and design of acute stroke trials.^17–19^ Although linear registration is a well-suited approach within a time point when there are no structural differences between the acquired images, it does not correct for distortion.^20^ In contrast, nonlinear registration corrects for distortion in follow-up imaging at 24 hours and 1 week in patients with stroke.^20^

This study investigates the use of nonlinear registration to a presenting magnetic resonance imaging (MRI) scan in the definition of IG in patients with acute ischemic stroke at 24 hours and 1 week. We use the mismatch of infarct volumes after linear and nonlinear registration to define AD, which is compared with preexisting methods. Given that MRI on presentation is not routine in clinical practice, alternative reference images to the presenting MRI in the definition of AD are evaluated. Exploratory analysis of the optimum volume of distortion at 24 hours to predict 11-mL distortion at 1 week is derived.^11^ Finally, the ability of distortion at 1 week to predict clinical deterioration was explored in this cohort.

## Methods

The data that support the findings of this study are available from the corresponding author on reasonable request.

### Patients

Patients aged >18 years with nonlacunar ischemic stroke were recruited within 18 hours of symptom onset into a prospective observational imaging cohort study under research protocols agreed by the UK National Research Ethics Service committee (ref 12/SC/0292 and 13/SC/0362) and by the local institutional review board. Written or witnessed consent was obtained from patients or agreement sought from a representative. Inclusion criteria were nonlacunar ischemic stroke and unilateral infarct visible on follow-up imaging at 24 hours, at 1 week, or both. Patients with a contraindication to MRI or impaired conscious level at presentation (score >1 on question 1a of the National Institutes for Health Stroke Scale) were not included. National Institutes for Health Stroke Scale was performed at the time of each scan. Six healthy volunteers were recruited and imaged under an agreed technical development protocol approved by the institution’s research governance office.

### Imaging

Patients were imaged on presentation using computed tomography (CT) and MRI as soon as possible after that. Follow-up MRI was performed the following day (24 hours) and at 3 to 9 days (1 week), whenever possible (Methods in the online-only Data Supplement). Healthy volunteers underwent T1-weighted MRI on 2 occasions, 1 week apart.

### Lesion Definition

All image analyses were performed using the Oxford Centre for Functional MRI of the Brain (FMRIB) software library (FSL). Lesion masks to define infarct at presentation were generated using the apparent diffusion coefficient (ADC) imaging and a threshold of 620×10^−6^ mm^2^/s (Methods in the online-only Data Supplement).^21^

All lesion masks used to define infarct at 24 hours and 1 week were defined manually by 2 separate independent stroke clinicians (4 different individuals across the study) using the masking tool in FSLView.^22^ The diffusion-weighted b1000 image (b=1000 s/mm^2^) was used for the 24-hour outcome^23^ and the T2-weighted fluid attenuated inversion recovery image for the 1-week outcome.^20^ Agreement was quantified using the concordance correlation coefficient. All lesion masks were reviewed and discrepancies resolved by a neuroradiologist. Masks were restricted to voxels within tissue masks created using the FMRIB’s automated segmentation tool (FAST).^24^

### Image Registration

Within-time point image registration was performed using linear (also known as rigid body) registration of either the diffusion-weighted or T2-weighted fluid attenuated inversion recovery images to the corresponding T1-weighted structural scan using FMRIB linear registration tool.^25,26^ Across time point, image registration of the follow-up T1-weighted image was made to the reference image space using both linear (FMRIB linear registration tool) and nonlinear registration using FMRIB’s nonlinear registration tool.^20,22^ Full details of registration are described in Methods in the online-only Data Supplement.

Infarct masks were resampled directly into the reference image space using a concatenation of the within-time point linear registration matrix, and either the nonlinear warp or the linear matrix generated from the registration of the T1-structural to the reference image. Once in the reference image space, the masks had a threshold of 0.5 applied.

### Infarct Growth

**Figure 1.**
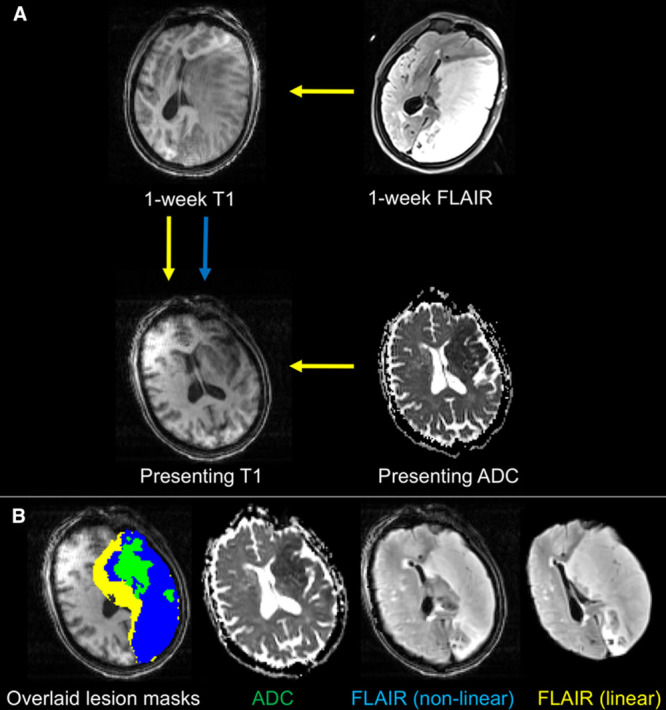
Schematic demonstrating the registration processes to quantify IG and presenting magnetic resonance imaging (MRI) distortion. **A**, The presenting apparent diffusion coefficient (ADC) lesion is registered to the presenting T1-weighted MRI using a linear registration to define infarct core. The T2-weighted (FLAIR) lesion mask at 1 week (or b1000 diffusion image at 24 hours) is registered to the presenting T1-weighted image using both linear (yellow arrows) and nonlinear (blue arrow) registration. **B**, The corresponding images registered to the presenting MRI are shown for reference. The difference between infarct core and the nonlinearly registered FLAIR lesion mask represents infarct growth (blue mask). The difference between the linear and nonlinear lesion masks represents anatomic distortion (yellow mask).

IG was defined at 24 hours and at 1 week. In keeping with the method most commonly used in stroke trials,^9^ uncorrected IG was calculated as the difference in volume between the follow-up infarct and the presenting ADC-defined lesion volumes. Corrected IG was calculated as the difference in volume between the follow-up infarct and the presenting ADC-defined lesion volumes after nonlinear registration to the presenting MRI (Figure 1).

Two of the stroke clinicians who defined infarction also scored the Alberta Stroke Program Early CT Score (ASPECTS) on all b1000 diffusion-weighted imaging MRI scans at a time independent of lesion definition, with discrepancies resolved by consensus. The change in ASPECTS score between scan time points was calculated to assess for IG into new anatomic territories.^11^ Corrected IG was compared between groups of patients categorized according to whether there was any deterioration in the ASPECTS.^10^

### Anatomic Distortion

AD was defined as a within-time point measure at 24 hours and at 1 week. AD is the difference in volume of the lesion mask volumes generated after linear and nonlinear registration to the reference image (Figure 1). Absolute AD was used as the primary measure because of the externally derived association of absolute edema volume (>11 mL) and poor long-term outcome.^11^ Relative AD (absolute AD relative to the final infarct volume) was also calculated to control AD for infarct size.

To quantify any systematic measurement error associated with AD, representative lesion masks (selected from the first [small] and third [large] quartiles of infarct volumes) were registered to the T1-weighted structural scans of 6 healthy volunteers who had been scanned on 2 separate occasions, 1 week apart. These representative infarcts are shown in Figure I in the online-only Data Supplement. The identical processing and analysis was followed to generate a pseudo MRI distortion volume as with the patient data.

Four different reference images were used for defining AD:

1. Presenting MRI distortion was considered as the benchmark comparator, given it is the closest approximation to the premorbid brain structure before the effects of edema and hemorrhage have manifested^6,20^;
2. Mirror MRI distortion was created by reflecting the follow-up structural MRI along the midline, using the patient’s own contralateral hemisphere as an approximation of an undistorted reference for the affected hemisphere;
3. Template MRI distortion was used as an external reference image with the 2-mm isotropic T1-weighted image in MNI152 space supplied with FSL as the standard^22,27,28^; and
4. Presenting CT distortion provided an alternative approximation to the premorbid brain structure, but relies on cross-modality registration and less spatial information with which to align anatomic regions.

All follow-up scans were reviewed for the presence of edema according to set criteria.^11^ Edema was defined as present if ≥2 of the following criteria were met on 2 axial slices: direct evidence of mass effect of affected gyri, indirect evidence based on new distortion of adjacent tissue, new midline shift, or new effacement of sulci or lateral ventricle. Any disagreements were resolved by consensus. The performance of AD was compared with this classification of the presence of edema.

A CSF-defined metric of AD was also calculated from the diffusion-weighted image (ADC maps) as described by Tipirneni-Sajja et al.^16^ In summary, CSF volume was quantified at presentation and follow-up, and the difference was used to estimate the degree of CSF displacement because of AD. Within each scan, CSF volumes were quantified using the ADC value of each voxel to estimate the proportion of CSF within that voxel using the formula: C=(ADC_voxel_−840)/2560, where ADC is measured in units of 10^−^^6^ mm2/s. The volumes of CSF in all voxels were then summed across the registered images to provide an estimate of total CSF volume.

### Analysis

Uncorrected and corrected IGs were correlated to explore the relationship between these metrics using Spearman correlation coefficient (*r*). The gradient of the correlation was calculated by linear regression and used to estimate the proportion by which uncorrected IG overestimated corrected IG. Corrected IG volumes were then compared across groups of patients with different changes in ASPECTS score from presentation to follow-up using ANOVA and the range of values of IG that exist within the groups described.

The median and interquartile range (IQR) of the absolute and relative pseudo MRI distortion within healthy volunteers were quantified. Median and IQR values of absolute and relative presenting MRI distortion were also calculated from within the stroke population at 24 hours and 1 week. Correlation of both absolute and relative presenting MRI distortion with infarct volume was calculated using Spearman correlation coefficient. Presenting MRI distortion volumes were compared between patients with and without rater-categorized edema using the Mann-Whitney *U* test.

The concordance correlation coefficient was used to quantify the agreement of presenting MRI distortion with CSF-defined distortion in all patients and separately for those with infarct volumes above and below the median.^29^ The grouping assessed the effect on small infarct volumes of the fixed error observed when using CSF-defined AD.^16^ Agreements of template MRI distortion, mirror MRI distortion, and presenting CT distortion with presenting MRI distortion were also quantified at 24 hours and 1 week using the concordance correlation coefficient from the patients where all metrics were available at both time points. Bland-Altman plots were used to explore the differences between presenting MRI distortion and other measures of AD.

The ability of the AD metric that had the highest agreement with presenting MRI distortion was evaluated as a tool at 24 hours to predict clinically significant AD at 1 week (11 mL)—a value derived in an external cohort of patients.^11^ Optimum thresholds were chosen using receiver operating characteristic (ROC) curve analysis followed by calculation of the Youden statistic.^30^ To explore the threshold of presenting MRI distortion in this cohort that predicted a clinical deterioration at 1 week, ROC curve analyses were performed using volumes of presenting MRI distortion at 1 week and any deterioration in National Institutes for Health Stroke Scale.

All statistical analyses were performed using Prism (GraphPad, CA) and Stata 15 (StataCorp LLC, TX).

## Results

### Patient Details

Of 57 consecutively enrolled patients, 37 met the criteria for inclusion, and patient demographics are presented in Table 1. Lack of follow-up imaging was the most common reason why patients did not meet the inclusion criteria for analysis: 9 patients for medical instability or death and 9 patients declined to undergo further MRI. Two patients had no lesion on imaging at follow-up. Thirty-six patients had a CT scan at presentation. All patients underwent MRI scanning at presentation (median delay CT to MRI, 1 hour 24 minutes; IQR, 54 minutes to 2 hours 11 minutes), 30 patients at 24 hours, 28 at 1 week, and 21 at all time points. Median ASPECTS were 7, 6, and 5 at presentation, 24-hour, and 1-week time points, respectively. Final infarct volumes ranged from 0.2 to 340 mL. The interrater concordance correlation coefficient was 0.99 at 24 hours and 0.98 at 1 week. Edema was categorized as present in 13 (43%) and 15 (52%) patients at 24 hours and 1 week, respectively. There was evidence of hemorrhagic transformation in 9 (29%, all hemorrhagic infarctions) and 7 (24%) scans at each time point. At 1 week and 24 hours, 6 and 5 patients exhibited both hemorrhagic transformation and edema. Midline shift was not seen within 24 hours of onset and in only 2 patients at 1 week.

**Table 1.**
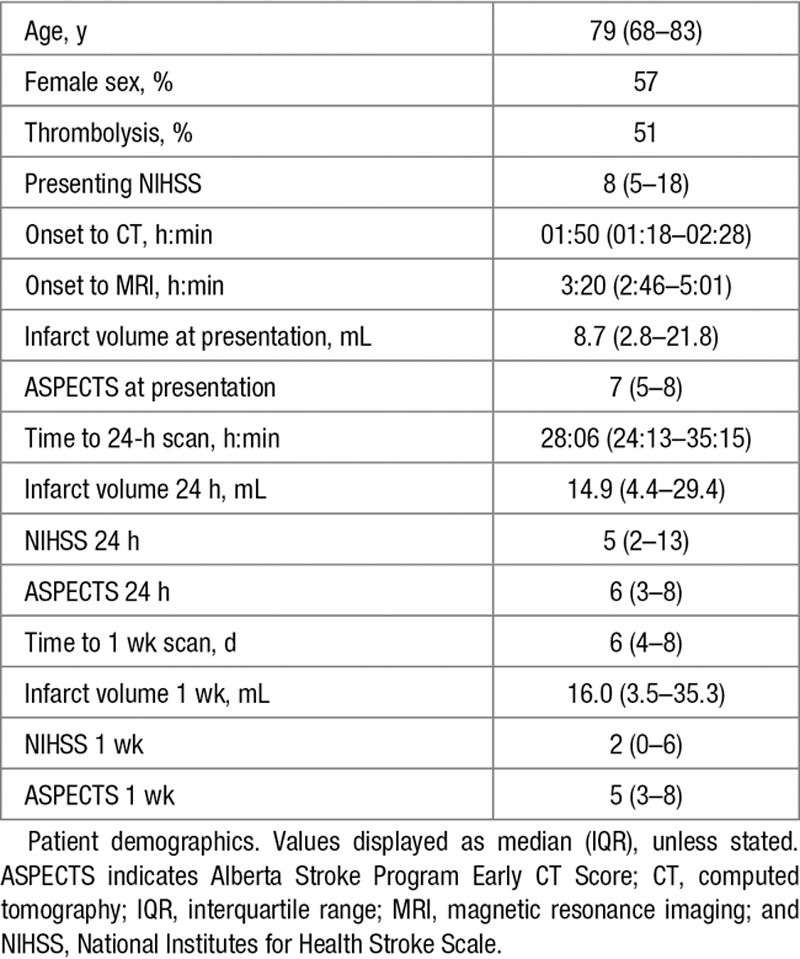
Patient Demographics

### Image Registration

**Figure 2.**
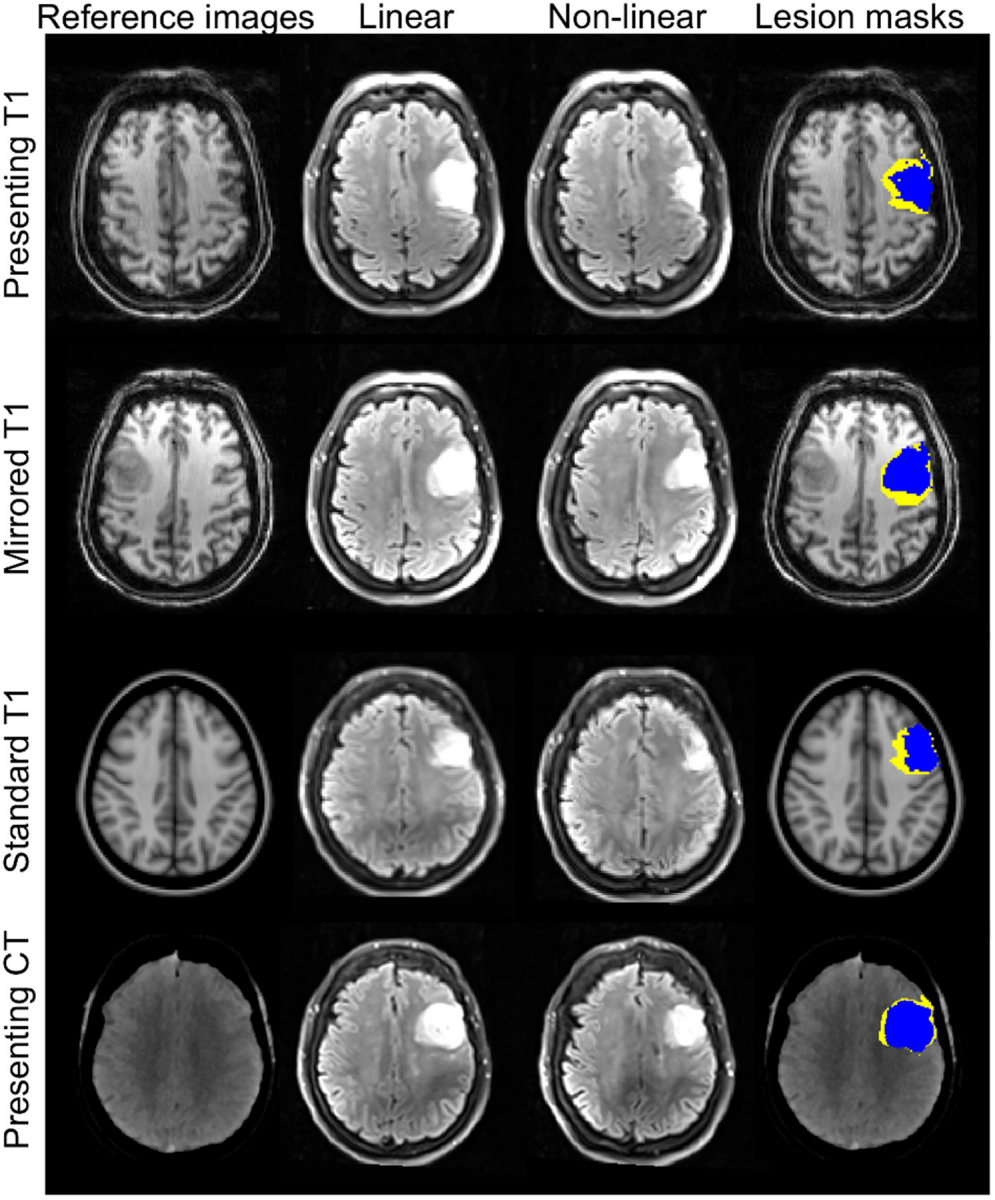
Representative imaging from a single patient. T2-weighted fluid attenuated inversion recovery imaging at 1 week and the associated infarct masks were registered to the different reference images. Imaging is displayed in the reference image space, accounting for the difference in image orientations. The anatomic distortion can be quantified as the difference between the linearly (yellow) and nonlinearly (blue) registered masks in the reference image space. CT indicates computed tomography.

The default registration algorithms were successful (example patient is shown in Figure 2) with the exception of the nonlinear registration of a 1-week scan from a single patient to its mirror T1-weighted image. In this case, the extensive infarct volume (340 plus 104 mL edema, presenting MRI distortion) resulted in insufficient unaffected brain with which to reference the mirror image. Registrations to presenting, template, and CT images were successful in all patients.

### Infarct Growth

Whereas corrected IG correlated strongly with uncorrected IG (*r*=0.98; *P*<0.0001) at both time points (Figure II in the online-only Data Supplement), uncorrected IG consistently overestimated corrected IG at both time points with gradients of 1.20 (95% confidence interval, 1.15–1.26) and 1.36 (95% confidence interval, 1.31–1.41) at 24 hours and 1 week, respectively. Corrected IG was not significantly different between patients grouped according to change in ASPECTS (*P*=0.1 and 0.2 at 24 hours and 1 week, respectively; ANOVA). Corrected IG of those patients with no change in ASPECTS over time was a maximum of 27.5 mL at 24 hours and 43.1 mL at 1 week.

### Anatomic Distortion

In healthy volunteers, the median absolute pseudo MRI distortion was −0.2 mL (IQR, −0.4 to −0.03) and −0.05 mL (IQR, −0.1 to −0.03) for large (32.5 mL) and small (9.2 mL) infarct volumes, respectively. The relative pseudo MRI Distortion values were −0.8% and −0.5% for large and small infarct volumes.

In patients with stroke, the median presenting MRI distortion values were 2.1 mL (IQR, 0.3–8.0) and 3.4 mL (IQR, 0.3–15.0) at 24 hours and 1 week, with median relative presenting MRI distortion values of 12% (IQR, 6%–23%) and 21% (IQR, 8%–29%). Absolute presenting MRI distortion values correlated with corrected infarct volume (Figure III in the online-only Data Supplement; 24 hours: *r*=0.85, *P*<0.0001; 1 week: *r*=0.94, *P*<0.0001), but relative presenting MRI distortion only correlated at 1 week (24 hours: *r*=0.32, *P*=0.1; 1 week: *r*=0.63, *P*=0.0004).

Presenting MRI distortion differed significantly between patients with and without rater-defined edema classification at both 24 hours and 1 week (median: 10.5 versus 0.3 mL, *P*<0.0001, and 14.4 versus 0.1 mL, *P*<0.0001, respectively; Mann-Whitney *U* test). Where the scan was classified as not having edema present, the maximum AD was 6.3 mL at 24 hours and 3.5 mL at 1 week. The presenting MRI distortion did not differ significantly between those with and without hemorrhagic transformation (*t* test; *P*=0.9).

**Figure 3.**
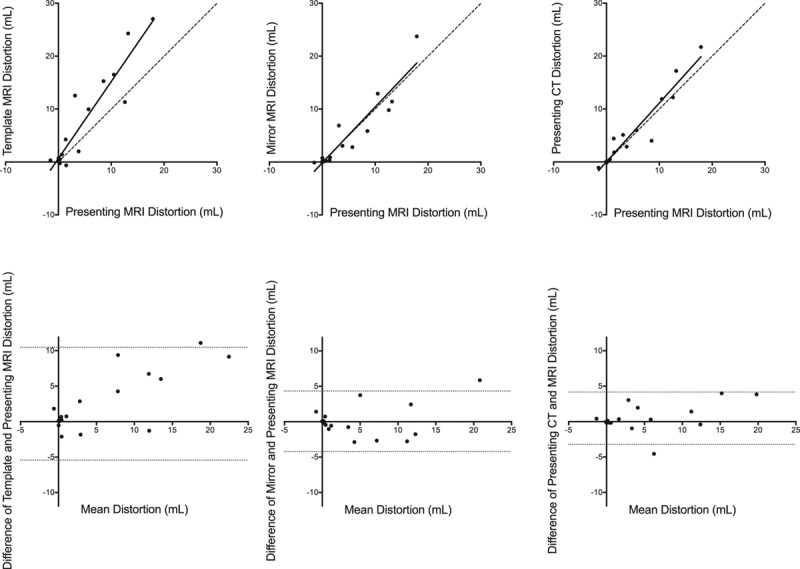
Agreement between registration-defined measures of anatomical distortion at 24 hours. **Top**, Template magnetic resonance imaging (MRI) distortion, mirror MRI distortion, and presenting computed tomography (CT) distortion compared with presenting MRI distortion at 24 hours. Continuous line: correlation; intermittent line: line of unity. **Bottom**, Bland-Altman plots showing the differences between the measurement techniques. Intermittent lines: 95% limits of agreement.

**Figure 4.**
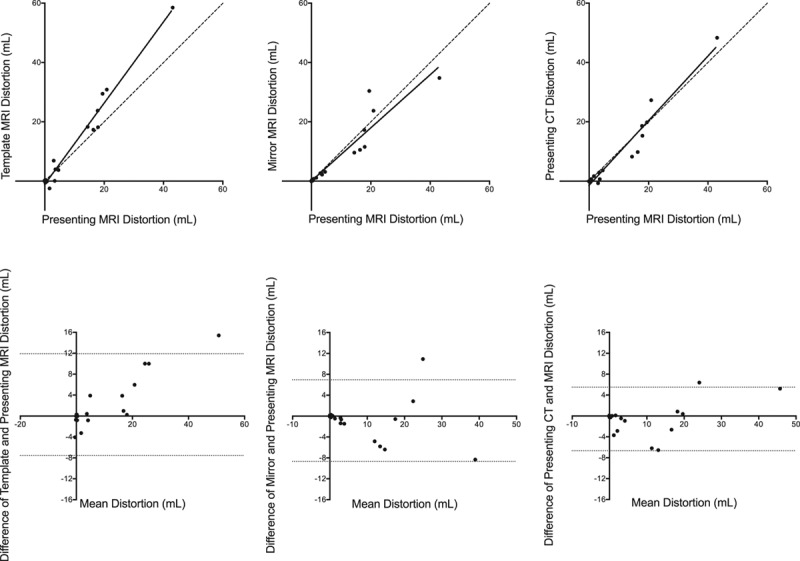
Agreement between registration-defined measures of anatomical distortion at 1 week. **Top**, Template magnetic resonance imaging (MRI) distortion, mirror MRI distortion, and presenting computed tomography (CT) distortion compared with presenting MRI distortion at 1 week. Continuous line: correlation; intermittent line: line of unity. **Bottom**, Bland-Altman plots showing the differences between the measurement techniques. Intermittent lines: 95% limits of agreement.

CSF-defined AD was concordant with presenting MRI distortion at 24 hours and 1 week (Table 2). However, there was no correlation for infarct volumes below the median volume at either time point. CSF-defined AD correlated with corrected lesion volumes similarly to presenting MRI distortion (Figure III in the online-only Data Supplement). There was greater concordance of absolute mirror MRI distortion, template MRI distortion, and presenting CT distortion with presenting MRI distortion at 24 hours and 1 week (Figures 3 and 4; Table 2). Representative images using all 4 reference approaches from 1 patient are shown in Figure 2. Presenting CT distortion displayed the strongest agreement at both time points with the benchmark comparator, presenting MRI distortion.

**Table 2.**
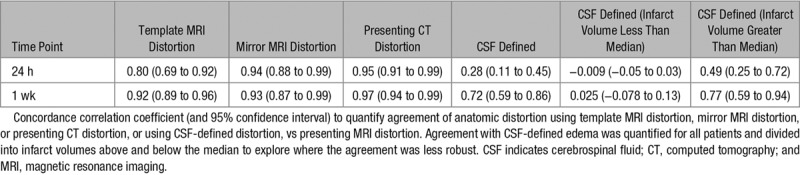
Agreement Between Measures of Anatomical Distortion

ROC curve analysis using presenting CT distortion at 24 hours to predict the externally derived, clinically meaningful threshold of distortion (11 mL) at 1 week generated an area under the curve of 0.99. The Youden statistic defined an optimum presenting CT distortion threshold of 4.8 mL at 24 hours for predicting edema of 11 mL at 1 week, with a sensitivity of 100% and a specificity of 93%. The CSF-defined metric of AD produced an area under the curve of 0.79 for the prediction of 11 mL edema at 1 week. Exploratory ROC curve analysis to derive a threshold of AD that predicted any increase in the National Institute for Health Stroke Scale from presentation to 1 week produced an area under the curve of 0.77 and an optimum threshold of 15 mL (Figure V in the online-only Data Supplement).

## Discussion

Using nonlinear registration to correct for AD provides not only improved estimates of IG but also quantifies the lesion expansion associated with edema and hemorrhagic transformation. AD can be quantified automatically across a range of infarct volumes in the absence of presenting MRI. Of the alternate reference images, the presenting CT scan provided the best comparator when compared against the benchmark presenting MRI. In this study, AD, when measured at 24 hours, could predict a clinically significant volume of AD at 1 week.

Lesion expansion over time comprises both IG and AD.^11^ In this study, uncorrected IG overestimated corrected IG by an average of 20% and 36% at 24 hours and 1 week. This overestimation represents the AD that occurs in the hours to days after stroke onset because of blood-brain barrier disruption that underlies both vasogenic edema and hemorrhagic transformation.^5–7^ That AD is a measure of edema is supported by the observation that AD is significantly different between patients with and without rater-categorized edema^11^ and, secondly, that presenting MRI distortion agreed with a similar measure of AD derived from CSF displacement.^16^

Absolute AD correlated more closely with infarct volume than relative AD. This points to the presence of individual factors that influence edema and hemorrhagic transformation—the absolute volumes of which may be proportional to the final infarct volume. It will require larger cohorts of patients to explore which clinical factors contribute to this variability. The error attributable to this registration-defined measure of AD was <1% when quantifying pseudo MRI distortion in healthy volunteers regardless of infarct volume used. This ability to measure small volumes of AD accurately is important because it provides the opportunity to estimate the threshold of AD at 24 hours that predicts clinically meaningful edema at 1 week (11 mL),^11^ which in this study was a 24-hour presenting CT distortion of 4.8 mL. Exploratory ROC curve analysis in this cohort showed that an optimum AD volume of 15 mL was most closely associated with clinical deterioration at 1 week, similar to the independently derived threshold of 11 mL.^11^

CSF-defined measures of distortion broadly agreed with registration-defined AD, but the relationship failed in patients with small (below median) infarct volumes. CSF-defined distortion is a global approach that measures CSF displacement within the whole skull. This limits its use in patients with smaller infarct volumes, not least because of errors ≤10 mL seen in patients with little or no evidence of infarction seen on MRI.^16^ This is in keeping with previously identified challenges of global measures of whole brain volume introduced by effects in regions remote from infarction, including noise, resorption of parenchymal extracellular fluid, and displacement of cerebral blood volume.^15,31^ In contrast, AD defined by nonlinear registration is a direct estimation of local distortion, and, therefore, any errors are likely to be proportional to the infarct volume.

Unlike presenting MRI distortion and CSF-defined distortion, template MRI distortion, mirror MRI distortion, and presenting CT distortion are not bound by the necessity to have an MRI scan at presentation. However, mirror MRI distortion could be adversely affected by the presence of a previous contralateral infarct, and the presence of global atrophy could adversely affect template MRI distortion. This latter effect may explain why template MRI distortion did not perform, as well as the other measures, given the heterogeneous degrees of global atrophy seen in a population of patients with stroke.

The data show a strong agreement between using the presenting CT scan with the presenting MRI as the benchmark on which to define AD. Cross-modality (CT to MRI) registration requires only a slight modification of the approach used for MRI-to-MRI comparison. This method could enable the comparison of CT-based ischemic core volumes with MRI-defined final infarction to quantify IG in addition to AD.^32,33^ This is an important area of future research given the fact that, on pragmatic grounds, CT imaging will be the core imaging modality on acute presentation in clinical trials. Furthermore, this approach can be incorporated into automated image analysis software to provide objective measures of IG and AD.

The differentiation of IG and AD provides the opportunity to test interventions aimed at either of these 2 specific processes in early-phase clinical trials. The same registration-based approach could also be implemented in preclinical stroke models, facilitating translation of novel approaches into early-phase trials.^34^ More consistent measurements improve statistical power in such studies, offering the potential to reduce sample size.^20^

This study has several limitations. Despite the minimal degree of measurement error seen in the pseudo MRI distortion metric, in a patient cohort, one might expect these errors of quantification to be greater. However, it is likely that any measurement error would remain proportional to the infarct volume. Further studies in larger cohorts are required to validate AD and IG derived from this registration approach by linking AD and IG to clinical outcomes and to explore the potential for defining IG using CT definitions of presenting infarct core. Limiting the immediate use of AD in historical clinical imaging cohorts is the requirement for a structural T1-weighted image acquired at the follow-up imaging time point.

## Conclusions

Registration-defined measures of IG and AD allow the distinction of the 2 processes of secondary injury that constitute lesion expansion. These techniques are objective, can be automated, and can be used to measure a wide range of infarct volumes.

## Acknowledgments

We wish to acknowledge the facilities and staff of the Oxford Acute Vascular Imaging Centre, the Oxford University Hospitals NHS Foundation Trust Acute Stroke Service, and the Acute Magnetic Resonance Imaging in Cerebral Ischemia group.

## Sources of Funding

This study was supported by the National Institute for Health Research (NIHR) Biomedical Research Centre, Oxford, the National Institute for Health Research Clinical Research Network, the Dunhill Medical Trust (grant No. OSRP1/1006), the Centre of Excellence for Personalized Healthcare funded by the Wellcome Trust and Engineering and Physical Sciences Research Council under grant number WT088877/Z/09/Z, the Oxford University Clinical Academic Graduate School, and the Wellcome Trust Institutional Strategic Support Fund (2014–2015).

## Disclosures

M. Jenkinson receives royalties from licensing of FSL to nonacademic, commercial entities. The other authors report no conflicts.

## Supplementary Material
