# Supplementary material for: Quantifying Infarct Growth and Secondary Injury Volumes: Comparing Multimodal Image Registration Measures
Source: Stroke. 2018 Jun 12;49(7):1647–55. doi: 10.1161/STROKEAHA.118.020788 (PMC6023577; doi:10.1161/STROKEAHA.118.020788)
Supplement: Supplementary file 1 [file str-49-1647-s001.pdf]

SUPPLEMENTAL MATERIAL

Contents

1. Supplementary Methods
2. Supplementary Figures

Supplementary Methods

Inclusion and exclusion criteria

Study inclusion criteria were: non-lacunar ischemic stroke; and, unilateral infarct visible on follow up imaging at 24 hours, at 1-week, or both. Patients with a contraindication to MRI, or impaired conscious level at presentation (score greater than 1 on question 1a of the National Institute for Health Stroke Scale (NIHSS)) were excluded.

Imaging protocol

Patients were imaged on presentation with a non-contrast 64-slice CT scan (GE Healthcare, Chicago, USA), and a MRI scan acquired as soon as possible after that. All MRI scans were obtained using a 3.0T Siemens Verio scanner (Siemens Healthcare, Erlangen, Germany). Scanning protocols included diffusion-weighted imaging (3 directions, 1.8×1.8×2.0mm, FoV=240mm, 4 averages, b = 0 and 1000s/mm2, TR = 9000ms, TE = 98ms, 50 slices, 2min 53sec) with apparent diffusion coefficient calculation; T1-weighted structural imaging (MPRAGE, 1.8×1.8×1.0mm, FoV=228mm, TR=2040ms, TE=4.55ms, 3min 58s) at all time points; and T2-weighted turbo spin echo FLAIR (1.9×1.9×2.0mm, FoV=240mm, TR=9000ms, TE=96ms) at 1-week.

Image Registration

Within timepoint image registration was performed using linear (also known as rigid body) registration of either the diffusion-weighted or T2-weighted FLAIR images to the corresponding T1-weighted structural scan using FMRIB's Linear Registration Tool (FLIRT; cost function: correlation ratio, six degrees of freedom).[1, 2]

Across timepoint image registration of the follow-up T1-weighted image was made to the reference image space using both linear (FLIRT) and non-linear registration using FMRIB's non-linear registration tool (FNIRT).[3, 4] Where the Presenting MRI, Mirror MRI, or CT scan was the reference image, linear registration with six degrees of freedom was used. When using the Standard MRI (MNI152 template) as the reference image,[4-6] a 12 degrees of freedom affine linear registration schedule was used to correct for differences in global dimensions. All registrations were checked visually for errors.

CT data were thresholded to a range of 0-100 Hounsfield units before isotropic resampling (1mm voxels) and application of a median filter. Brain extraction was then performed using the FSL Brain Extraction Tool (BET).[7, 8] T1-weighted structural image intensities were transformed in order to flatten them and match the intensities of the CT images. More specifically, a 4-class tissue segmentation of the T1-weighted image was performed and then the contrast between the normal appearing grey matter and normal appearing white matter was removed, to match the lack of contrast in the CT images. The remaining intensity differences (e.g., between CSF and normal appearing grey matter) were left untouched. This flattening allowed the cost function implemented within the non-linear registration tool in FSL (FNIRT) - sum of squared differences together with a smooth multiplicative bias field - to be validly applied to the image pair (CT and flattened T1-weighted MRI). Without the flattening, the non-linear registration is suboptimal due to the mismatch in contrast, which this cost function is unable to model.

CT data were registered to the follow up T1-weighted structural scan with these flattened intensities using FLIRT and FNIRT as described above. Once registered, the linear transformation matrix and the non-linear warps were inverted to provide the inputs for MRI to CT registration used in the analysis.

Infarct masks were resampled directly into the reference image space using a concatenation of the within time point linear registration matrix, and either the non-linear warp or the linear matrix generated from the registration of the T1-structural to the reference image. Once in the reference image space, the masks had a threshold of 0.5 applied.

Presenting lesion definition

Lesion masks to define infarct at presentation were generated using the ADC imaging and a threshold of 620×10^{-6} mm^2/s.[9] Cluster-based analysis was performed on these original masks with the maximum volume cluster automatically identified and smoothed with a kernel of standard deviation of 1mm.[10] Cluster analysis was repeated with automated selection of the maximum cluster volume, which was then restricted to the original masks to minimize inclusion of any suprathreshold voxels as a result of smoothing. The automated masks were inspected to ensure the cluster or clusters correctly represented the acute infarct. Any errors were manually corrected, either by removing noise, or reselecting the correct cluster.

1. Jenkinson M, Bannister P, Brady M, Smith S. Improved optimization for the robust and accurate linear registration and motion correction of brain images. *Neuroimage*. 2002;17:825-841

2. Jenkinson M, Smith S. A global optimisation method for robust affine registration of brain images. *Medical image analysis*. 2001;5:143-156
3. Harston GW, Minks D, Sheerin F, Payne SJ, Chappell M, Jezzard P, et al. Optimizing image registration and infarct definition in stroke research. *Ann Clin Transl Neurol*. 2017;4:166-174
4. Jenkinson M, Beckmann CF, Behrens TE, Woolrich MW, Smith SM. FSL. *Neuroimage*. 2012;62:782-790
5. Mazziotta J, Toga A, Evans A, Fox P, Lancaster J, Zilles K, et al. A four-dimensional probabilistic atlas of the human brain. *J Am Med Inform Assn*. 2001;8:401-430
6. Mazziotta JC, Toga AW, Evans A, Fox P, Lancaster J. A probabilistic atlas of the human brain: theory and rationale for its development. The International Consortium for Brain Mapping (ICBM). *Neuroimage*. 1995;2:89-101
7. Muschelli J, Ullman NL, Mould WA, Vespa P, Hanley DF, Crainiceanu CM. Validated automatic brain extraction of head CT images. *Neuroimage*. 2015;114:379-385
8. Smith SM. Fast robust automated brain extraction. *Human brain mapping*. 2002;17:143-155
9. Purushotham A, Campbell BC, Straka M, Mlynash M, Olivot JM, Bammer R, et al. Apparent diffusion coefficient threshold for delineation of ischemic core. *Int J Stroke*. 2015;10:348-353
10. Smith SM, Brady JM. SUSAN - A new approach to low level image processing. *Int J Comput Vision*. 1997;23:45-78

Supplementary Figure I

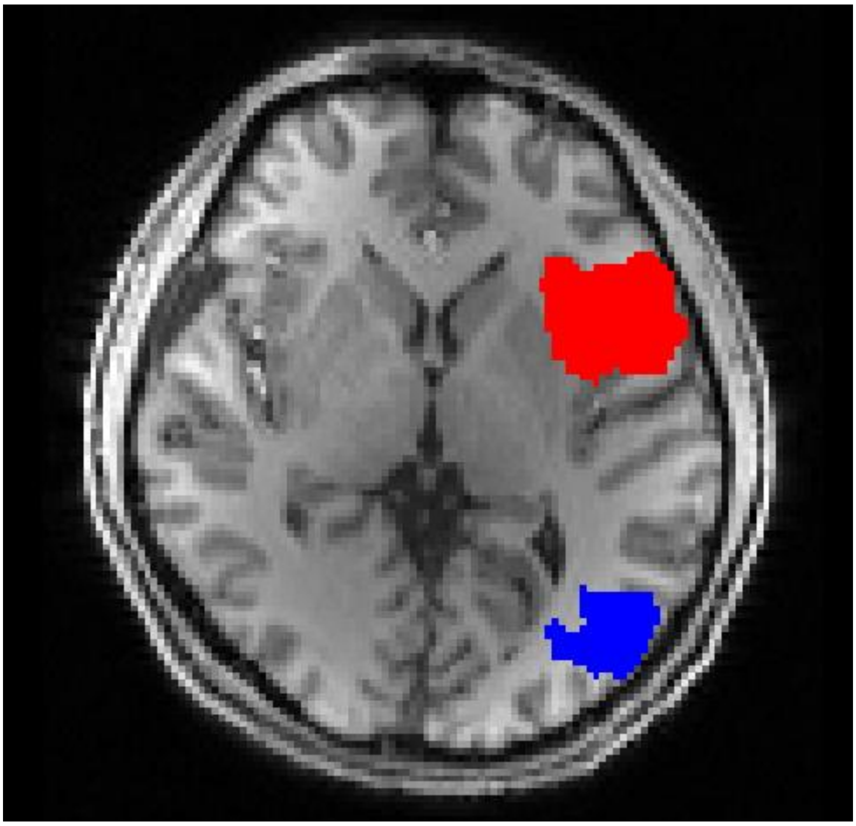

Example of infarct masks taken from the first (small, blue) and third (large, red) quartile of stroke lesions in the stroke cohort registered to the T1-weighted image of a healthy volunteer.

Supplementary Figure II

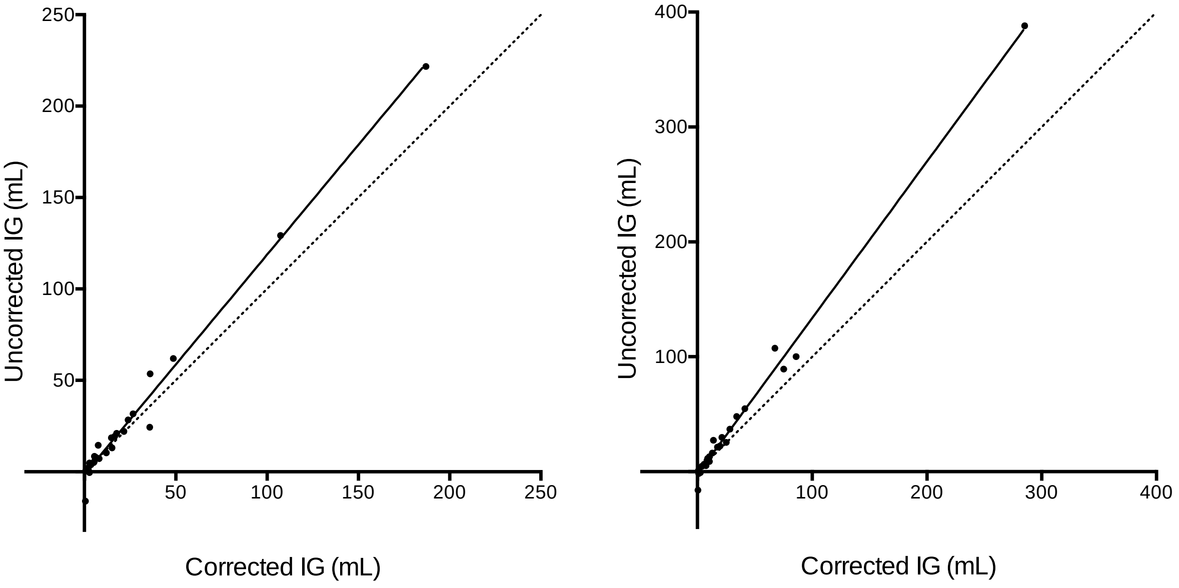

Uncorrected infarct growth (IG) plotted against Corrected IG at 24-hours (left panel) and 1-week (right panel). Uncorrected IG consistently overestimates Corrected IG with correlation gradients of 1.20 (1.15-1.26, 95% confidence interval) and 1.36 (1.31-1.41) at 24-hours and 1-week respectively. Continuous lines are the linear regressions and interrupted lines are lines of unity for reference.

Supplementary Figure III

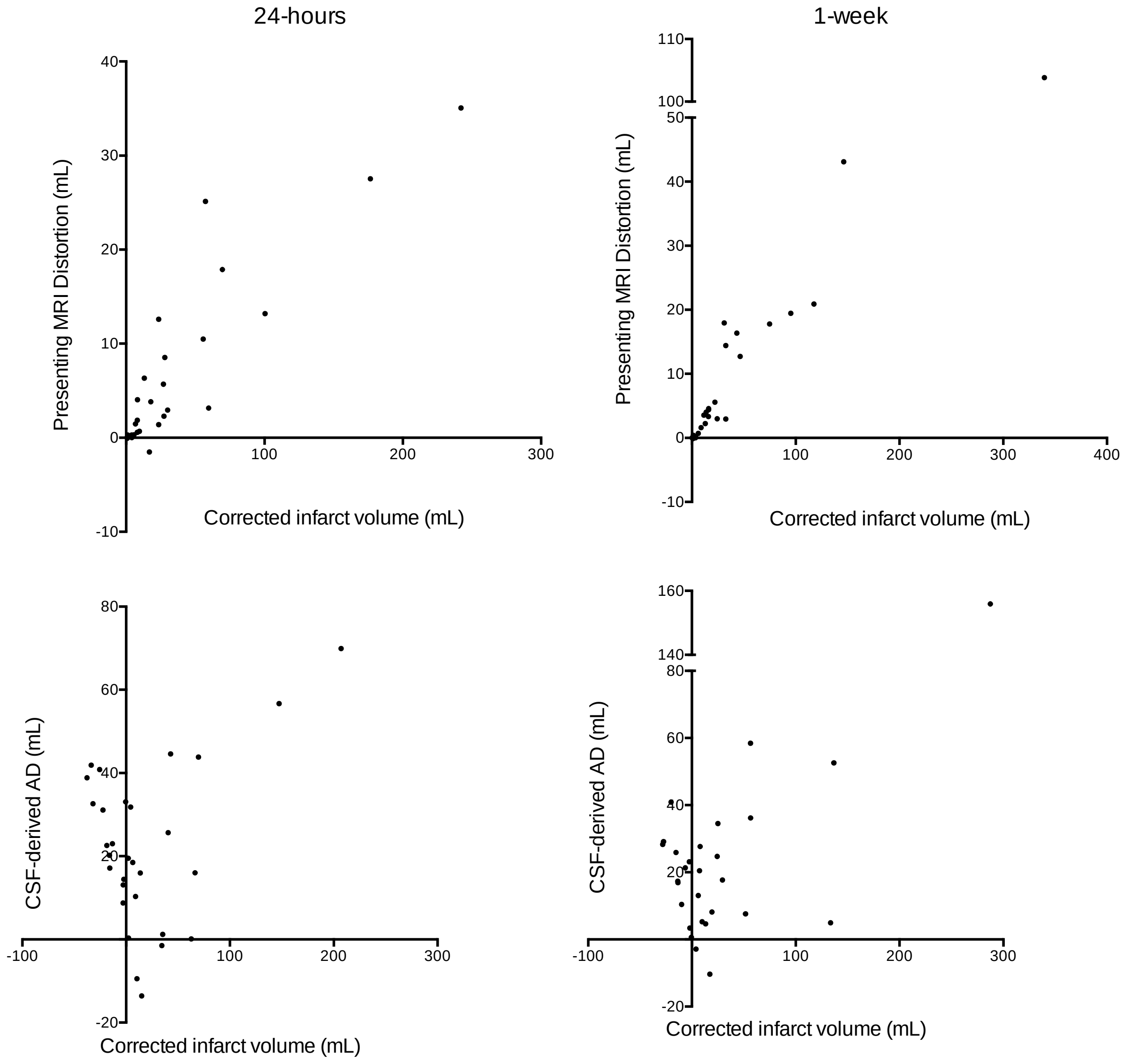

Absolute quantifications of edema correlated with corrected infarct volumes at 24-hours (left panels) and 1-week (right panels). The upper panels show Presenting MRI Distortion plotted against infarct volume corrected by registration, and lower panels show CSF-defined edema against infarct volume corrected by CSF distortion.

Supplementary Figure IV

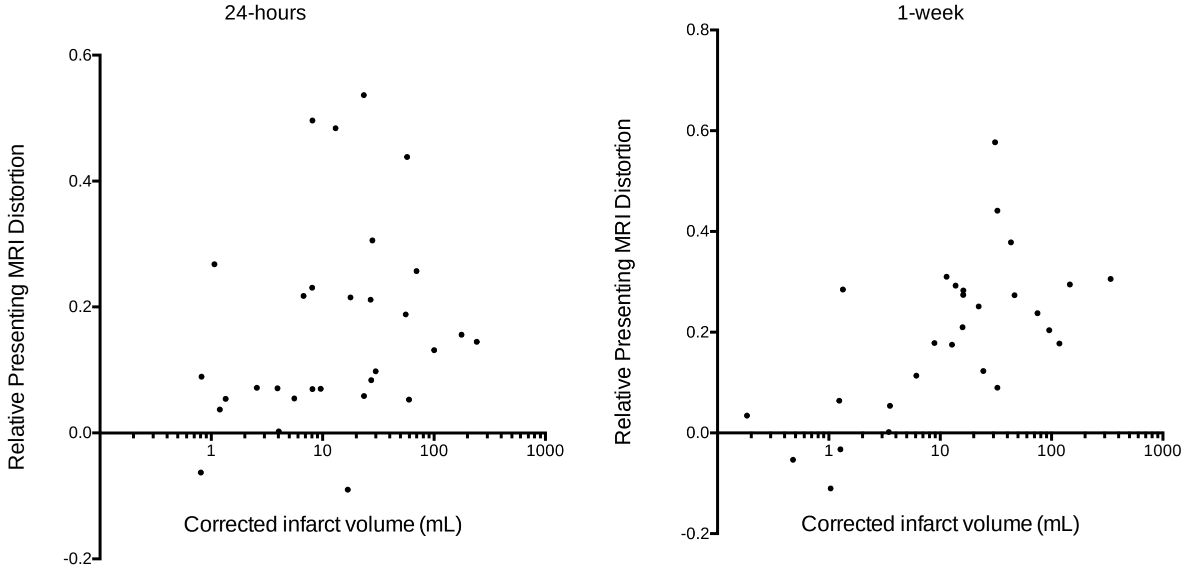

Relative quantifications of edema correlated with corrected infarct volumes at 24-hours (left panel) and 1-week (right panel). The graphs show relative Presenting MRI Distortion plotted against infarct volume corrected by registration on a logarithmic scale.

Supplementary Figure V

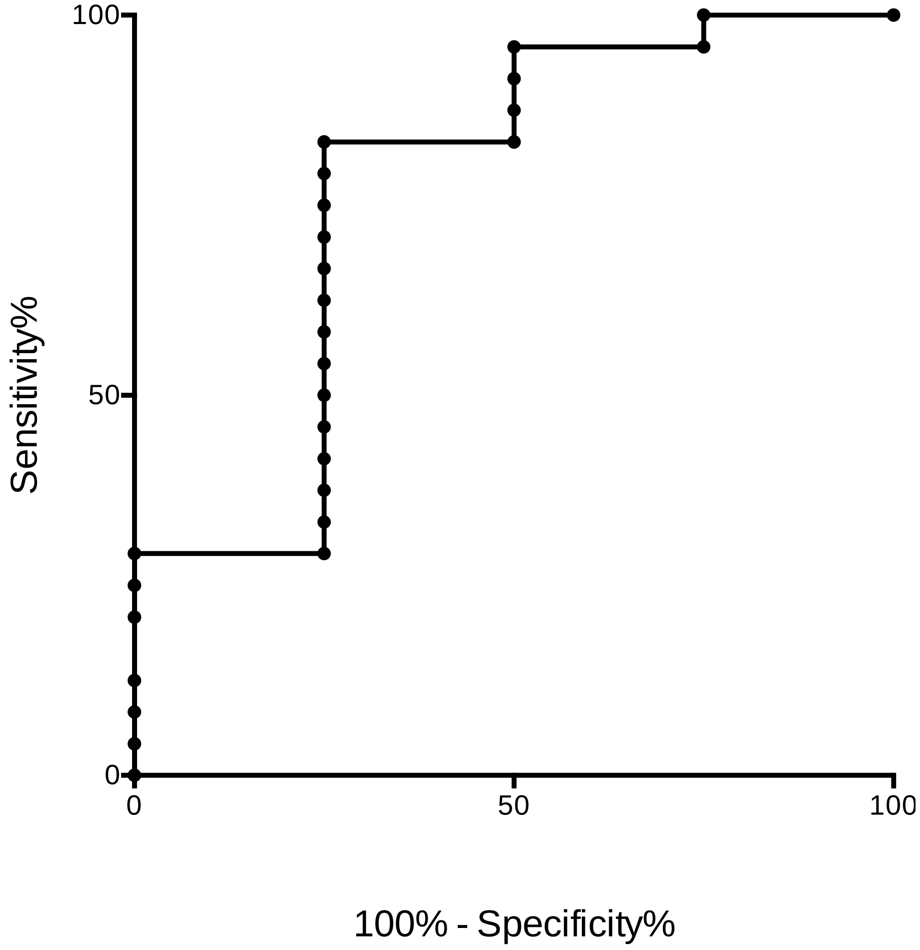

Receiver operating characteristic curve of Presenting MRI Distortion at 1-week to predict increase in National Institute for Health Stroke Scale at 1-week. Area under the curve = 0.77.
